# Safety, Immunogenicity, and Vaccine Compatibility of a Trivalent Inactivated In Ovo Nanovaccine Against Avian Colibacillosis in Broilers Under Commercial Hatchery Conditions

**DOI:** 10.3390/ani16060931

**Published:** 2026-03-16

**Authors:** Angelo Scuotto, Daniela Ogonczyk-Makowska, Romain Magnez, Bryan Thiroux, Pierre-Louis Herrouin, Thomas Bouillet, Anaïs-Camille Vreulx, Amélie Degraeve, Didier Betbeder

**Affiliations:** Vaxinano SAS, 84 Rue du Dr. Yersin, 59120 Loos, France; angelo.scuotto@vaxinano.com (A.S.); daniela.ogonczyk@vaxinano.com (D.O.-M.); romain.magnez@vaxinano.com (R.M.); bryan.thiroux@vaxinano.com (B.T.); pierre-louis.herrouin@vaxinano.com (P.-L.H.); t-p.bouillet@vaxinano.com (T.B.); anaiscvreulx@gmail.com (A.-C.V.); amelie.degraeve@vaxinano.com (A.D.)

**Keywords:** colibacillosis, broiler, in ovo, inactivated vaccine, nanoparticle, *E. coli*

## Abstract

Avian colibacillosis, caused by *Escherichia coli*, remains a significant threat to poultry health and production, particularly in the context of rising antimicrobial resistance. Efficient and scalable vaccination strategies are needed to reduce economic losses and reliance on antibiotics. This study investigated the safety and immunogenicity of a novel single-dose in ovo vaccine candidate based on three inactivated *E. coli* strains formulated with cationic maltodextrin nanoparticles. The vaccine was evaluated in broilers under commercial hatchery conditions. In ovo administration was well tolerated and did not adversely affect hatchability, survival, growth performance, or feed efficiency. Vaccinated birds mounted a measurable serum immunoglobulin Y (IgY) response against *E. coli* from 14 days post-hatch, which persisted until slaughter age. Furthermore, when co-administered with routinely used live-attenuated viral vaccines, no interference with the immunogenicity of these vaccines was observed. These results demonstrate that the inactivated nanovaccine is safe, immunogenic, and compatible with an industrial-scale in ovo vaccination. The findings support its potential as a practical prophylactic approach to prevent avian colibacillosis in broiler production.

## Simple Summary

Avian colibacillosis is a major cause of economic losses in poultry production worldwide. Control of the disease is increasingly challenging due to rising antibiotic resistance, highlighting the need for effective vaccination strategies suitable for large-scale use. In this study, we evaluated an in ovo nanovaccine candidate against avian colibacillosis in broilers based on three inactivated *Escherichia coli* strains that are associated with cationic maltodextrin nanoparticles. The vaccine was assessed under commercial conditions in a Brazilian broiler hatchery. In ovo administration of the vaccine was well tolerated and did not negatively affect hatchability, survival, growth performance, or feed efficiency. Vaccinated birds developed a measurable antibody response against *E. coli* from 14 days post-hatch, which persisted until slaughter age. Importantly, the vaccine did not interfere with the immune response to other routinely used in ovo live-attenuated viral vaccines. These results support the potential of this inactivated nanovaccine as a safe and practical preventive approach against avian colibacillosis in commercial broiler production systems.

## 1. Introduction

Avian Pathogenic *E. coli* (APEC), a major agent of colibacillosis, is a pathogen that affects poultry production worldwide. The disease affects broilers, laying hens, as well as breeding chickens and turkeys, causing significant losses in the industry [1]. Along with uropathogenic and neonatal meningitis-associated *E. coli*, it is classified as extraintestinal *E. coli* (ExPEC) and shows a zoonotic potential of causing disease in humans [2]. Rather than being characterized by a specific genotype, APEC are a collection of different pathotypes, often belonging to the O1, O2 and O78 serotypes [3,4]. Although APEC are characterized by the presence of many virulence-associated genes (VAGs), none of them have been described to be their exclusive characteristics [5], and 18% of APEC that are isolated from dead birds from broiler farms are not characterized [6]. This high genetic diversity is one of the challenges to the development of successful colibacillosis prevention strategies.

Although *E. coli* is a normal member of the natural gut microbiota of birds, it can become dangerous when found outside of the digestive tract [7,8], particularly in the respiratory tract, which is closely associated with other organs in birds due to the absence of a diaphragm separating the thoracic and abdominal cavities. APEC can act as a primary or secondary pathogen, causing diseases in birds that are weakened, stressed, or infected by another pathogen, which is commonly either Newcastle disease virus (NDV) or infectious bronchitis virus (IBV) [1]. The most common cause of infection is the inhalation of bacteria. APEC spreads to the lower respiratory airways, ultimately inducing the inflammation of the air sacs and adjacent tissues. Ingestion of feed contaminated by feces can also lead to infection. Vertical transmission between laying hens and egg production has also been documented [4]. The most common symptoms of colibacillosis are airsacutitis, perihepatitis, pericarditis and myocarditis, omphalitis (yolk sac infection), peritonitis, cellulitis, swollen-head syndrome, and femoral head necrosis, leading to high rates of mortality and carcass condemnation [1,9,10].

First-week mortality is an important parameter for measuring the quality of broiler production [11], and bacterial infection is the cause of 53.5% of deaths [12]. *E. coli* and *Enterococcus* spp. have been identified in 28.7 and 19.4% of death cases, respectively, and mixed infection of those two bacteria is found in 24.8%. A study by Kemmet et al. showed that of all birds that died in the first week of life, 70% showed symptoms of colibacillosis [13]. Overall, APEC infections cause 1–10% mortality in chickens [14,15]. The economic losses due to colibacillosis in the poultry industry are estimated at hundreds of millions of dollars every year [16,17].

Traditionally, colibacillosis-preventive strategies include biosecurity measures [18], antibiotic administration, and vaccinations. With the increasing problem of antibiotic resistance, the use of antibiotics has become restricted, highlighting the need for alternative preventive strategies. Vaccines, along with pre- and probiotics or phage therapy, can reduce reliance on antibiotics by preventing infections rather than treating them [19,20]. Vaccinations provide long-term protection against specific pathogens for NDV, infectious bursal disease virus (IBDV), and Marek’s Disease Virus (MDV) and are strongly recommended or even mandatory in poultry production [21]. Currently, commercial vaccines against colibacillosis that are available on the global market rely on different formulation strategies, such as live attenuated strains and inactivated subunit preparations. Live attenuated vaccines (LAVS) are typically based on the *E. coli* O78 serotype, which is attenuated through specific genetic deletions, such as the *aroA* or *crp* virulence genes, and are generally administered via coarse spray or drinking water. While these vaccines deliver a wide array of bacterial antigens and can induce potent humoral and cellular immune responses, they are associated with several biological, safety, and practical constraints, including the potential risk of reversion to virulence, limited cross-protection against heterologous serotypes, possible interference with concurrent treatments or vaccinations, regulatory and biosafety concerns requiring careful handling, reduced stability compared to inactivated formulations, and safety considerations in immunocompromised or stressed flocks. In contrast, other available options include inactivated subunit vaccines, such as those consisting of purified *E. coli* fimbrial (F11) and flagellar (Ft) antigens. These are typically administered via intramuscular injection between 6 and 12 weeks of life. In broiler production, however, this timeframe does not grant protection during the most vulnerable weeks that occur immediately post-hatching. Furthermore, subunit vaccines present a more limited number of *E. coli* antigens compared to whole-cell approaches, and parenteral administration also makes the vaccination highly labor-intensive in large-scale broiler farms. In addition, such formulations are often adjuvanted with liquid paraffin, which can significantly influence the production cost per dose. Inactivated vaccines are also commonly administered after the second week post-hatching, which is after the peak period of colibacillosis-related mortality in broilers [11]. Autogenous *E. coli* vaccines, prepared from farm-specific field isolates, are widely used in poultry production. However, their application in commercial broiler farms is limited because of the short production cycle and logistical constraints, making commercial standardized vaccines more practical in this context.

Consequently, the poultry industry still lacks a colibacillosis vaccine that combines high safety, broad antigenic coverage, and ease of administration at a low price per dose. The in ovo administration route was explored for the first time for delivering Marek’s disease vaccines in the early 1980s [22]. It provides an efficient way for a standardized and automated vaccination of hundreds of eggs at the same time, which is in line with the intensification of poultry production. The advantages of this mass vaccination method are numerous, and it is no surprise that it is becoming increasingly popular. Today, 90% of industrial broiler hatcheries in the United States employ in ovo vaccinations to immunize the broilers [23]. Commercial in ovo vaccines are available against Marek’s Disease, NDV, IBDV, fowlpox, and coccidiosis, and they are most often based on live attenuated pathogens [24].

Cationic maltodextrin nanoparticles with a lipidic core (NPL) were previously presented as a flexible and highly customizable platform for antigen delivery and were used in the context of nasal vaccination against toxoplasmosis, leishmaniasis, and influenza [25]. *Toxoplasma gondii* purified inactivated parasites (PIP) were encapsulated within NPL and evaluated across various species, including mice [26,27], sheep [28], and 58 species of exotic animals, including marsupials, felids and non-human primates [29], proving effective in preventing *T. gondii* infection, and showing potential for therapeutic effect [30]. Similar results were obtained when vaccinating mice and treating infected dogs with Leishmania infantum PIP [31,32]. Inactivated bacterial vaccines offer several advantages. They circumvent the risk of inducing disease since the pathogen is killed. Furthermore, their production is simpler and safer than live-attenuated vaccines (LAVs), which reduces the need for highly specialized facilities. Additionally, the storage and stability of inactivated vaccines are simpler than live vaccines.

A new inactivated nanovaccine against colibacillosis was developed for in ovo administration. Briefly, NPL were used to deliver the antigens of three inactivated *E. coli* strains, namely O1:K1:H7, O78:K80, and O2:K1:H6. The safety, immunogenicity, and compatibility of co-administration with live attenuated vaccines after in ovo injection in the poultry industry were assessed in a large-scale industrial hatchery.

## 2. Materials and Methods

### 2.1. Confirmation of Strain Identity by PCR

The identity of the three *E. coli* strains used in this study—NCTC 9001 (O1:K1:H7), NCTC 11101 (O2:K1:H6), and NCTC 9078 (O78:K80:H-)—was confirmed by the PCR targeting the serotype-specific O-antigen gene clusters. Genomic DNA was extracted from bacterial colonies using the InstaGene matrix (Bio-Rad Laboratories, Hercules, CA, USA), following the manufacturer’s protocol. Briefly, three to four individual colonies were resuspended in 1 mL of DNase/RNase-free ultrapure water and centrifuged at 12,000 rpm for 1 min. The pellet was resuspended in 200 µL of InstaGene matrix, incubated at 56 °C for 30 min, then at 100 °C for 8 min. After centrifugation at 12,000 rpm for 3 min, the supernatant was collected and used as a DNA template.

Three specific primer pairs were used to amplify strain-discriminatory fragments of the wzy or wzx genes involved in O-antigen biosynthesis:

- For NCTC 9001 (O1), primers wzyO1-Fw1/wzyO1-Rv1 amplified a 778 base pair (bp) fragment.
- For NCTC 11101 (O2), primers wzxO2-Fw3/wzxO2-Rv3 amplified a 684 bp fragment.
- For NCTC 9078 (O78), primers O78-Fw2/O78-Rv2 amplified a 582 bp fragment.

Each PCR was performed in a total volume of 25 µL, containing 12.5 µL of DreamTaq Green PCR Master Mix (Thermo Fisher Scientific, Waltham, MA, USA), 1 µL of each primer (10 µM), 0.5 µL of nuclease-free water, and 10 µL of extracted DNA. The thermocycling protocol was: initial denaturation at 95 °C for 3 min; 35 cycles of denaturation at 95 °C for 30 s, annealing at 55 °C for 30 s, and extension at 72 °C for 1 min; and a final extension step at 72 °C for 10 min. The PCR products were analyzed on a 2% agarose gel stained with GelRed (GelRed, Biotium, Hayward, CA, USA), using a 100 bp DNA ladder.

### 2.2. Detection of APEC Virulence-Associated Genes

The presence of five key virulence genes commonly associated with Avian Pathogenic *E. coli* (APEC)—*iroN*, *ompT*, *hlyF*, *iss*, and *iutA*—was evaluated by multiplex PCR. DNA was extracted from bacterial pellets that were obtained from bioreactor-grown cultures, inactivated, and harvested as described previously, using an InstaGene matrix. After incubation at 56 °C and thermal lysis at 100 °C, supernatants were collected following centrifugation (12,000 rpm, 3 min) and used directly as a PCR template.

Each multiplex PCR reaction (25 µL) contained 12.5 µL of DreamTaq Green PCR Master Mix (Thermo Fisher Scientific, Waltham, MA, USA), 0.2 µL of each primer (40 µM) for all five targets (final concentration 0.32 µM per primer), 0.5 µL of nuclease-free water, and 10 µL of a DNA template (diluted to 10 ng/µL). Thermocycling conditions were as follows: initial denaturation at 94 °C for 2 min; 30 cycles of 94 °C for 30 s, 63 °C for 30 s, and 72 °C for 1 min; and a final extension at 72 °C for 10 min. The PCR products were separated on a 2% agarose gel, run at 70 V for 4 h, and stained with GelRed. The band sizes were compared with a 100 bp DNA ladder.

### 2.3. Antigen Preparation

Three pathogenic *E. coli* strains, O1:K1:H7 (NCTC 9001), O78:K80 (NCTC 9078), and O2:K1:H6 (NCTC 11101), were purchased from the National Collection of Type Cultures of the UK Health Security Agency, London, UK. Each strain was produced separately in a sterile 500 mL spinner-flask (Corning, Avon, France) for 24 h at 37 °C. The bacteria were then transferred to a 20 L bag (8 L working volume, Flexsafe optical, Sartorius, Aubagne, France) and incubated for 24 h at 37 °C. The bacterial suspension was then centrifuged, the pellet was washed with phosphate-buffered saline (PBS, Thermo Fisher Scientific, Waltham, MA, USA) and was inactivated overnight with 0.3% formaldehyde at 37 °C. The bacterial pellet was then washed thrice with sterile water and stored at −80 °C until the vaccine formulation. The inactivation of the bacteria and the sterility of the antigen preparation were confirmed by plating the inactivated bacteria suspension on agar plates (Tryptic soy agar or Sabouraud Dextrose agar) and observing no bacterial or fungal growth after a 48 h incubation at 37 °C.

### 2.4. Vaccine Preparation

NPLs were produced following good manufacturing practices (GMP), as previously described [33]. The process involved dissolving maltodextrin in NaOH under magnetic stirring at room temperature. Epichlorohydrin (Alfa Aesar, Schiltigheim, France, cat A15823.22) and GTMA (Sigma Aldrich, Saint-Quentin-Fallavier, France, cat 50053-50ML) were subsequently added to form a dense, cationic gel, which was then processed through a high-pressure homogenizer and ultrafiltered to remove residual salts. Lipidation with 70% of Dipalmitoylphosphatidylglycerol (Lipoid, Les Ulis, France, cat 560300-2150040) by weight was performed, yielding the final NPL solution.

The in ovo vaccine was prepared by combining the NPL with inactivated APEC at 1:0.3, 1:0.5 or 1:1 antigen-to-NPL weight ratio in water and at room temperature under stirring. Hereafter, the inactivated APEC antigens formulated with NPL at antigen-to-NPL weight ratios of 1:0.3, 1:0.5, or 1:1 are collectively referred to as VXN-EColi. The formulation was stored at 4 °C until use. Each vaccine dose consisted of a total of 100 μg of APEC antigens (33,33 µg of each one of the three strains), as quantified using a Bicinchoninic acid assay (Thermo Fisher Scientific, Waltham, MA, USA), and a corresponding quantity of NPL, delivered by Newxxitek™ HVT + ND (Zoetis, Kalamazoo, MI, USA), and BDA BLEN (MSD Animal Health, Rahway, NJ, USA) vaccines in a total volume of 50 µL in ovo injection. Based on the antigen-to-NPL ratios tested (1:0.3, 1:0.5 and 1:1), the corresponding NPL quantities were 33 µg, 50 µg and 100 µg per dose, respectively.

### 2.5. Vaccination

In a commercial hatchery, 4800 fertile Ross eggs were divided into four groups of 1000 and incubated for three weeks. Three days before the hatching (D-3), each egg from all groups was injected with two mandatory poultry vaccines: a bivalent vaccine against MDV and NDV-Newxxitek™ HVT + ND, which were administered jointly with a monovalent vaccine against IBV-BDA BLEN. G1 received Newxxitek™ HVT + ND and BDA BLEN only. Other groups received the two vaccines in combination with VXN-Ecoli at three different ratios of *E. coli* antigens-to-NPL: 1:0.3 (G2), 1:0.5 (G3) or 1:1 (G4) (Table 1). The vaccinations were performed using EMBREX INOVOJECT^®^ NXT (Zoetis, Kalamazoo, MI, USA) in an ovo vaccination machine, delivering a 50 µL injection of the three vaccines into the amniotic sac of each egg. After the hatching, 350 broilers per group were randomly selected for each group in the study, transported and housed in 1.2 m^2^ isolators at less than 10 kg/m^2^ at Imunova Análises Biológicas LTDA (Curitiba, Brazil) animal facility, and fed ad libitum with a commercial broiler diet formulated according to the standard nutritional requirements and the recommendations for their age. All animal experiments were evaluated and approved by the Ethics Committee for Animal Research of Imunova Análises Biológicas, protocol number 06/2021.

### 2.6. Sample Collection and Analysis

**Figure 1:**
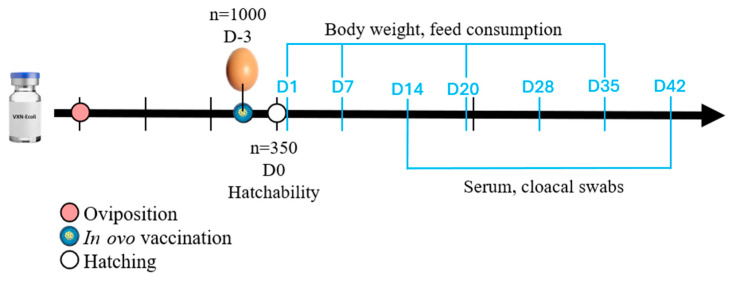
An experimental timeline. The data collection points are marked with blue lines.

Cloacal swab and blood collection were performed at D14 (*n* = 22) and D42 (*n* = 22). The zootechnical performance parameters (weight gain, feed intake, corrected feed conversion, and mortality) of 350 broilers from each group were assessed on D1, D14, D28, and D42. The experimental timeline is shown in Figure 1.

### 2.7. Zootechnical Performance

Each experimental group included 350 birds, a number chosen to reliably monitor zootechnical performance. Average feed intake was calculated as the difference between the weight of the feed provided and the weight of the leftovers at the end of each period. Feed conversion ratio (FCR) was calculated according to standard poultry practice, as the ratio of feed intake to total flock weight increases over each period. Mortality rate was expressed as the difference between the number of birds housed at the beginning and at the end of the study.

### 2.8. IgA and IgY Quantification

*E. coli* LPS-specific IgY production was evaluated with an enzyme-linked immunosorbent assay (ELISA) protocol. The samples were diluted in 1% casein in PBS. The ELISA plates were coated with field strain *E. coli* Lipopolysaccharide (LPS) at 2 µg/mL in PBS and incubated overnight at 4 °C. The plates were washed three times with 200 µL/well of PBS supplemented with 0.05% Tween20 (Sigma-Aldrich, St. Louis, MO, USA) and let soak for 5 min/wash. Wells were blocked with 1% casein in PBS for 1 h at room temperature (RT). The samples were tested in a serial dilution. The plates were washed as described above, and an anti-chicken IgY (BioRad Laboratories, Hercules, CA, USA) diluted in PBS supplemented with 0.1% casein was added at a 1:5000 dilution for 1 h at RT. After washing, the assay was developed with 3,3,5,5-Tetramethylbenzidine (TMB) single solution (Life Technologies, Waltham, MA, USA) for 15 min at RT in the dark, and the reaction was stopped by adding 50 µL of 1 M sulfuric acid to each well. Absorbance was measured at 450 nm. *E. coli*-specific IgA production was assessed using the same protocol, with an anti-chicken IgA detection antibody at a 1:2000 dilution (Bio-Rad Laboratories, Hercules, CA, USA). Commercial Idexx ELISA kits were used to quantify serum IgY antibodies against NDV (IDEXX NDV Ab Test for chickens, cat 99-09263, Westbrook, ME, USA) and IBDV (IDEXX IBD-XR Ab Test, cat 99-09261, Westbrook, ME, USA) viruses. Within each group, a subset of 22 birds was randomly selected for immunological analyses.

Cloacal swabs were collected from each bird at D14 and D42 using sterile swabs, which were immediately placed into 1 mL of phosphate-buffered saline (PBS) containing 0.05% Tween 20 and 0.5% bovine serum albumin (BSA). The swabs were vortexed briefly, and the supernatants were collected after centrifugation at 3000× *g* for 10 min at 4 °C. The resulting supernatants were used for IgA detection. Anti-LPS IgA antibodies in cloacal swabs were measured by indirect ELISA using the same coating and detection conditions as for serum IgY, with modifications for mucosal samples: sample volumes were adjusted to 100 µL per well, and incubation times were increased to 90 min at room temperature to improve antigen–antibody binding. The optical density was measured at 450 nm, and titers were calculated as the reciprocal of the highest dilution yielding an OD value above the mean + 2 SD of the negative control wells.

### 2.9. Statistical Analysis

The experimental unit was defined as an individual bird. The data visualization and statistical analysis were conducted using GraphPad Prism 8.3. The raw data was analyzed to identify and exclude outliers using the ROUT method, with exclusion criteria based on a predetermined false discovery rate (FDR) of Q = 1. Each dataset underwent the D’Agostino–Pearson, Anderson–Darling, Shapiro–Wilk and Kolmogorov–Smirnov tests to assess normality, comparing data distribution to a Gaussian model. The data that met the normality criteria were analyzed using a conventional ANOVA test followed by Dunn’s post hoc test. The data that did not meet normality assumptions were analyzed using the Kruskal–Wallis test followed by Tukey’s post hoc correction. The results with a *p*-value less than 0.05 were considered statistically significant.

## 3. Results

### 3.1. Strain Confirmation by O-Antigene Gene PCR

The serotype identity of the three reference *E. coli* strains—NCTC 9001 (O1:K1:H7), NCTC 11101 (O2:K1:H6), and NCTC 9078 (O78:K80:H−)—was validated by the PCR amplification of serogroup-specific O-antigen biosynthesis genes. Distinct amplicon sizes were obtained for each strain using dedicated primer sets designed from reference O-antigen gene cluster sequences (GU299791.1 for O1, GU299792.1 for O2, and KJ778787.1 for O78).

NCTC 9001 yielded a single amplicon of 778 bp using the wzyO1 primer pair. NCTC 11101 produced a 684 bp product with the wzxO2 primers. NCTC 9078 generated a 582 bp amplicon with the O78-specific primer set.

**Figure 2:**
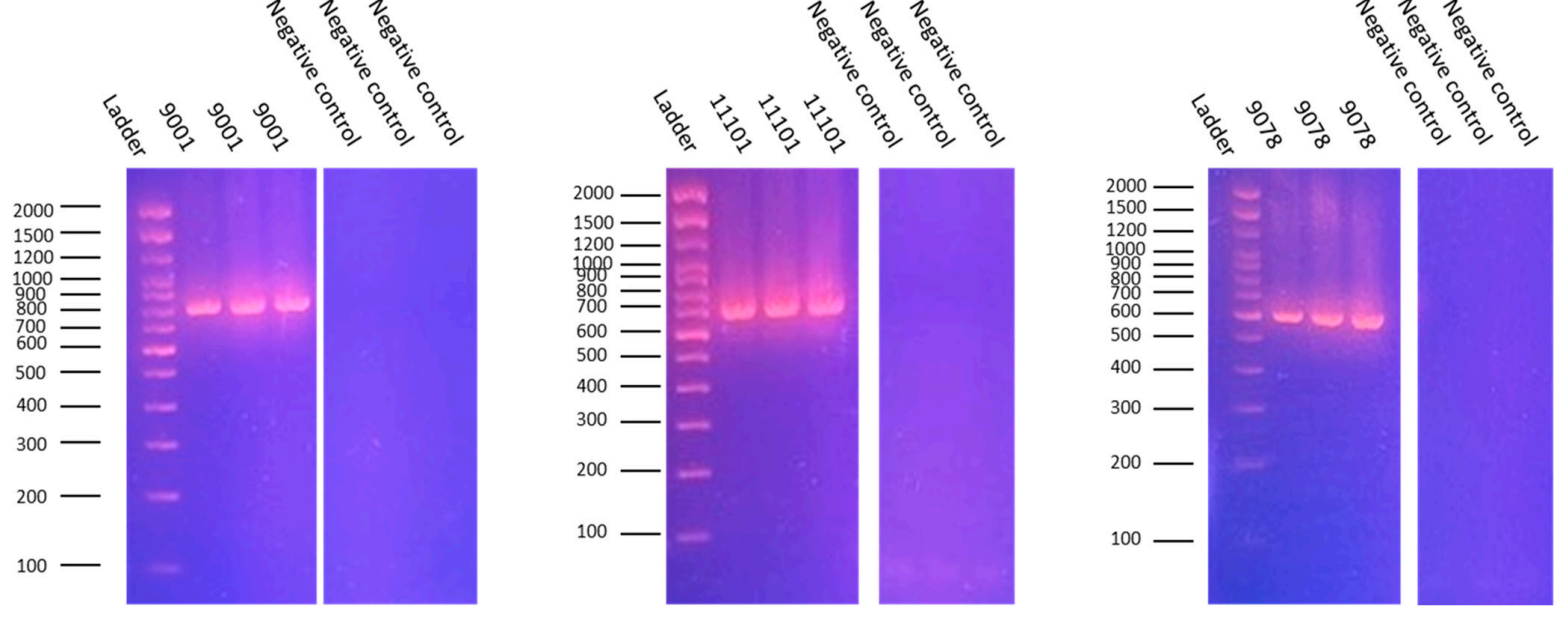
Confirmation of strain identity by the PCR targeting O-antigen genes. The agarose gel electrophoresis of the PCR products using serogroup-specific primers targeting the O-antigen gene clusters in three reference *E. coli* strains: NCTC 9001 (O1:K1:H7), NCTC 11101 (O2:K1:H6), and NCTC 9078 (O78:K80:H−). Each strain produces a single distinct amplicon of the expected size (778 bp, 684 bp, and 582 bp, respectively), confirming accurate serogroup identification. No amplification was detected in no-template controls (NTCs), demonstrating assay specificity.

Each strain was tested in triplicate to ensure reproducibility and to confirm assay specificity. Identical amplification profiles were observed across replicates. No non-specific bands were detected, and all of the no-template controls remained negative (Figure 2)

### 3.2. Detection of APEC Virulence-Associated Genes

The amplicon sizes corresponded to the expected lengths: 553 bp *(iroN*), 496 bp (*ompT*), 450 bp (*hlyF*), 323 bp (*iss*), and 302 bp (*iutA*). NCTC 9001 (O1:K1:H7) tested positive for all five virulence genes. NCTC 11101 (O2:K1:H6) was positive only for *iroN*. NCTC 9078 (O78:K80:H−) carried only the *iutA* gene.

**Figure 3:**
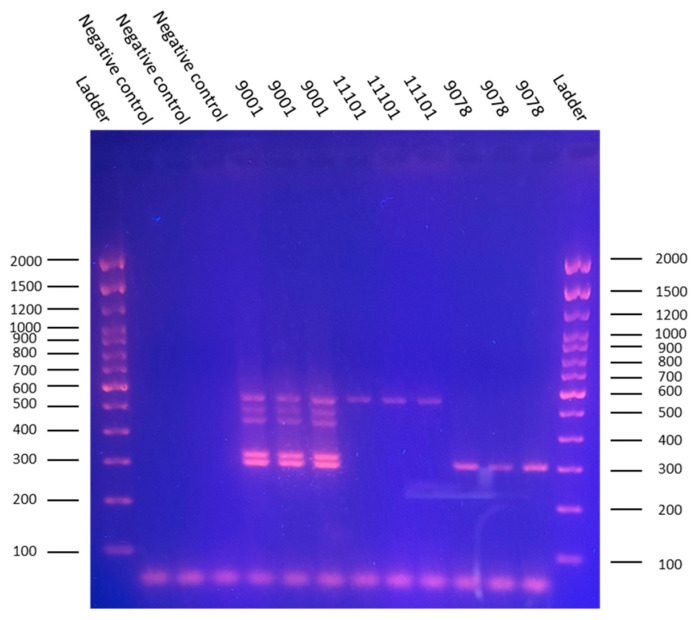
The multiplex PCR detection of APEC virulence-associated genes in *E. coli* strains. The multiplex PCR profiles showing the detection of five virulence genes (*iroN*, *ompT*, *hlyF*, *iss*, and *iutA*) in the three reference strains. Strain NCTC 9001 harbors all five genes, indicating a broad virulence potential. Strain NCTC 11101 carries only *iroN*, and NCTC 9078 possesses only *iutA*. The amplicon sizes correspond to expected lengths, and no amplification in negative controls confirms assay specificity.

Agarose gel electrophoresis confirmed the specificity of the multiplex PCR, with minimal background and no false-positive signals (Figure 3).

### 3.3. Safety and Zootechnical Performance Following In Ovo Vaccination with VXN-EColi

Hatchability rates of fertile eggs ranged from 94.13% to 95.82% across all experimental groups and were not significantly affected by the addition of VXN-EColi at any antigen-to-NPL ratio (Table 2). The average egg weights at the time of injection were also comparable between groups, indicating homogeneous starting conditions prior to in ovo vaccination.

Post-hatch survival was high in all groups, with survival rates at D42 ranging from 94.29% to 96.96% (Table 3). No statistically significant differences in mortality were observed between vaccinated and control groups, and no dose-dependent effect associated with increasing NPL content was detected. Overall, these results indicate that an in ovo administration of VXN-EColi, regardless of the antigen-to-NPL ratio, did not adversely affect hatchability or post-hatch survival.

**Figure 4:**
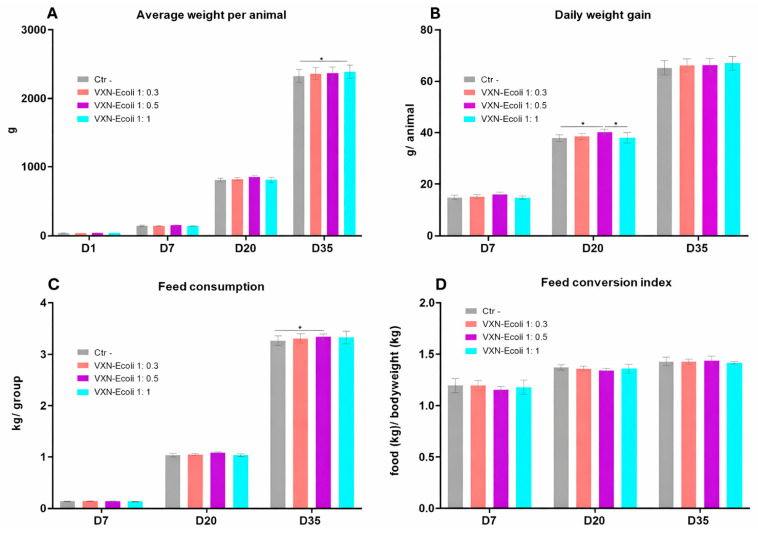
The zootechnical performance of the vaccinated broilers. (**A**) The average weight per animal and (**B**) the average daily weight gain per animal, calculated as the ratio between the average weight of broilers in each group and the age of the birds in days. (**C**) The feed consumption per group and (**D**) the feed conversion index, calculated as a ratio of the feed consumed by the flock and its total weight. The outliers were identified by the ROUT test (Q = 1%) and eliminated from the analysis. The graphs present means with 95%CI. The data on graphs (**A**–**C**) was analyzed using one-way ANOVA followed by a post hoc Tukey multiple comparisons test, and the differences were not statistically significant. The data on graph (**D**) was analyzed using a two-way ANOVA followed by post hoc Tukey multiple comparisons tests. The statistical significance is indicated as follows: * *p* < 0.05.

The zootechnical performance was monitored at four time points during the rearing period (Figure 4). Overall, the broilers vaccinated with VXN-EColi exhibited growth performances that were largely comparable to the negative control group throughout the experiment. The average body weight increased progressively over time in all groups, with no significant differences observed between treatments, except at D35, where broilers vaccinated with VXN-EColi 1:1 displayed a significantly higher mean body weight compared to the negative control (Figure 4A).

The daily weight gain followed a similar pattern across groups (Figure 4B). A transient increase was observed at D20 in the VXN-EColi 1:0.5 group, which showed a significantly higher weight gain compared to the VXN-EColi 1:1 and the negative control groups, while no differences were detected at the other time points. The feed consumption increased over time in all groups and was slightly but significantly higher in the VXN-EColi 1:0.5 group at D35 compared to the negative control (Figure 4C). Despite these variations, the feed conversion ratio remained comparable between groups at all time points (Figure 4D), indicating that vaccination did not adversely affect feed efficiency.

### 3.4. Compatibility of Inactivated VXN-EColi with Live Antiviral Vaccines

**Figure 5:**
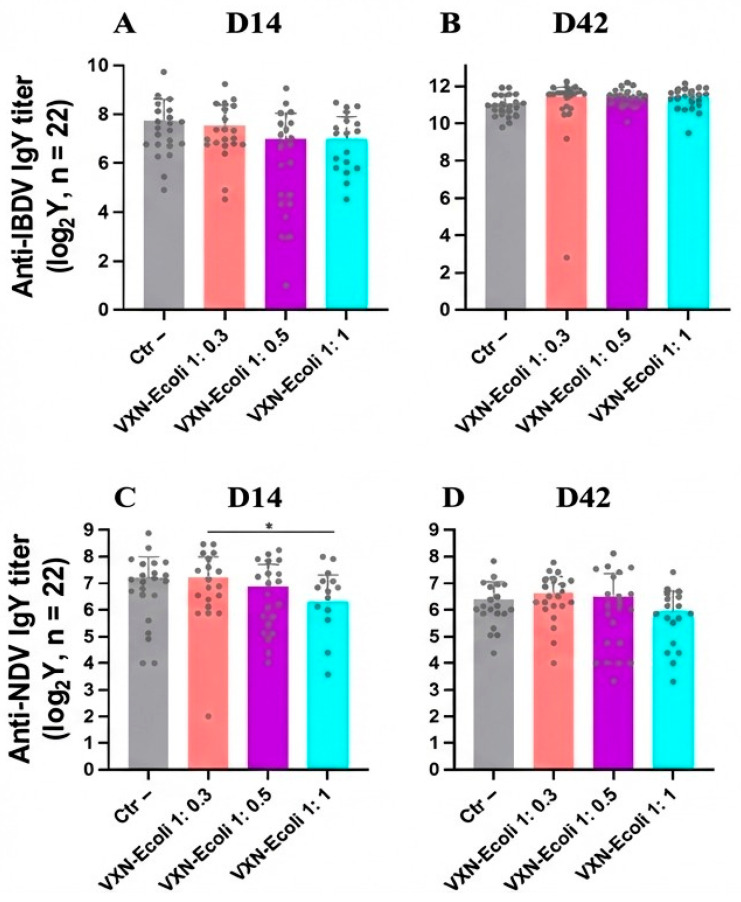
The levels of antiviral IgY antibodies against IBDV (**A**,**B**) and NDV (**C**,**D**) in the serum of the broilers were measured by ELISA at D14 and D42. The results represent the highest serum dilution that produces a positive signal and are presented as means with 95%CI on a logarithmic scale. The outliers were identified by the ROUT test (Q = 1%) on raw data and eliminated from the analysis. The data was analyzed after removing the outliers using the Kruskal–Wallis test followed by Dunn’s multiple comparisons tests (NDV at D42 and IBDV at D14) or a one-way ANOVA followed by Tukey’s multiple comparisons tests (NDV at D14 and IBDV at D42). The statistical significance is indicated as follows: * *p* < 0.05.

The impact of the VXN-EColi vaccination on the immunogenicity of the Newxxitek™ HVT + ND and BDA BLEN vaccines was evaluated by measuring the serum IgY titers against NDV and IBDV. No statistically significant differences in NDV- or IBDV-specific antibody titers were observed between groups vaccinated with VXN-EColi and the negative control group (Figure 5).

### 3.5. Humoral Immune Response Induced by VXN-EColi Vaccination

**Figure 6:**
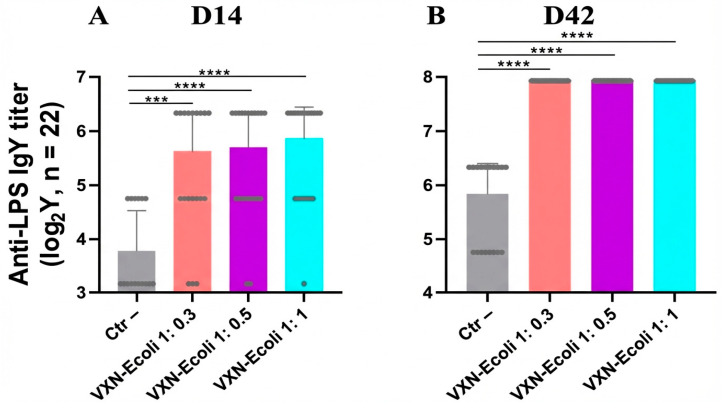
The anti-LPS IgY titers measured in serum at D14 (**A**) and D42 (**B**) were determined by ELISA. The antibody titers are expressed as endpoint dilutions corresponding to the highest sample dilution yielding a positive signal and are presented on a logarithmic scale as means with 95%CI. The outliers were identified on raw data using the ROUT test (Q = 1%) and excluded from the analysis. The data were analyzed independently for each time point using Kruskal–Wallis tests followed by Dunn’s multiple comparison tests. The statistical significance is indicated as follows: *** *p* < 0.001, **** *p* < 0.0001.

**Figure 7:**
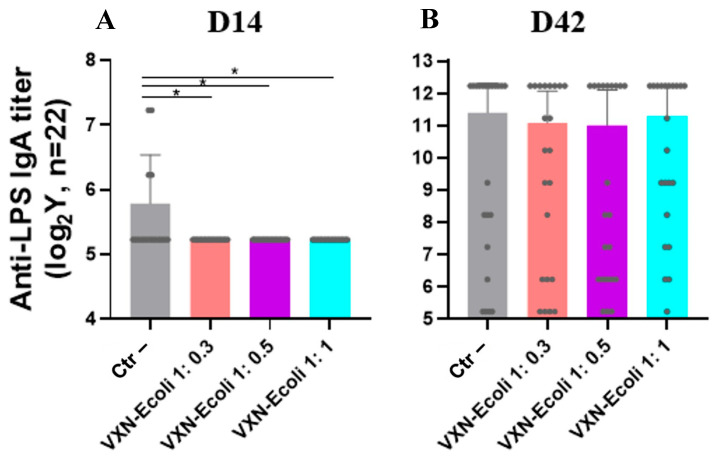
The anti-LPS IgA titers measured in cloacal swabs at D14 (**A**) and D42 (**B**) were determined by ELISA. The antibody titers are expressed as endpoint dilutions corresponding to the highest sample dilution yielding a positive signal and are presented on a logarithmic scale as means with 95% confidence intervals. Each dot represents an individual animal (*n* = 22 per group prior to outlier exclusion). The outliers were identified on raw data using the ROUT test (Q = 1%) and excluded from the analysis. The data were analyzed independently for each time point using Kruskal–Wallis tests followed by Dunn’s multiple comparison tests. The statistical significance is indicated as follows: * *p* < 0.05.

The anti-LPS IgY and IgA antibody titers were measured respectively in a serum, and the cloacal swabs were collected at D14 (Figure 6A and Figure 7A) and D42 (Figure 6B and Figure 7B). A statistically significant increase in serum anti-LPS IgY titers was observed in all VXN-EColi-vaccinated groups at both D14 and D42 compared to the negative control. At D14, serum IgY titers increased 3.49-, 3.71-, and 4.38-fold in the VXN-EColi 1:0.3, 1:0.5, and 1:1 groups, respectively. At D42, all vaccinated groups reached an identical 4.26-fold increase relative to controls. No significant differences in serum IgY titers were observed between the different antigen-to-NPL ratios at either time point. No increase in anti-LPS IgA titers was detected in the cloacal swabs from VXN-EColi-vaccinated groups compared to the negative control. The highest IgA titers were consistently observed in the negative control group at both D14 and D42. The statistical analyses using Kruskal–Wallis tests followed by Dunn’s multiple comparison tests indicated no significant differences between the negative control and any vaccinated group at D14 or D42 (all *p* > 0.05). All groups reached similar peak IgA titers at D42, with no significant differences between antigen-to-NPL ratios.

## 4. Discussion

The in ovo administration of vaccines represents a critical advancement in poultry production, offering a synchronized and automated approach to early-life immunity. However, the success of this route is often limited by the delicate balance between inducing a potent immune response and maintaining embryonic viability [34].

The PCR-based characterization of the reference strains confirmed the purity and identity of the O1, O2, and O78 serogroups. However, the multiplex PCR revealed significant genomic diversity in their VAG profiles. While the O1 (NCTC 9001) strain harbored the complete set of tested VAGs (*iroN*, *ompT*, *hlyF*, *iss*, and *iutA*), the O2 and O78 strains displayed more restricted profiles. This heterogeneity reflects the complex landscape of APEC pathotypes in the field and underscores the necessity of a trivalent approach to provide broad antigenic coverage.

A critical barrier to in ovo vaccination is the potential for embryonic toxicity or post-hatch growth retardation [35,36]. Our data show that VXN-EColi, even at the highest nanoparticle (NPL) ratio, did not impair hatchability or early survival rates. Throughout the 42-day rearing period, feed conversion ratios (FCR) and daily weight gains remained comparable to those of the control groups. Interestingly, at D35, the group vaccinated with the 1:1 ratio showed a significant weight increase compared to controls. This suggests that the NPL-based formulation is not only non-toxic but may also promote optimal growth, possibly by reducing the subclinical bacterial burden early in life, a hypothesis that warrants further investigation through challenge studies.

VXN-EColi induced a robust and sustained systemic humoral response, with a significant 4.26-fold increase in serum anti-LPS IgY titers at slaughter age (D42). The absence of a dose-dependent effect between the different antigen-to-NPL ratios suggests that even lower concentrations of nanoparticles are sufficient to adjuvant the inactivated bacterial antigens effectively, which is encouraging for optimizing production costs in large-scale operations. In contrast, no vaccine-induced increase in cloacal IgA was observed at D14 (Figure 7). The higher IgA levels in the control group likely reflect natural exposure and colonization by commensal or field *E. coli* strains during rearing [37,38]. By D42, all groups showed elevated IgA titers, but the variability was high, and no significant differences were observed between the vaccinated and control groups. This variability may result from differences in the sampling technique, dilution of mucosal secretions, and the relatively low abundance of anti-LPS IgA in cloacal swabs. As the assay was adapted from serum IgY detection and is not fully standardized for mucosal samples, these results should be interpreted with caution.

These findings indicate that the in ovo delivery of NPL-formulated inactivated antigens primarily stimulates systemic immunity. This effect is likely mediated by the properties of the nanoparticles: cationic maltodextrin particles interact with cell membranes, promoting uptake by antigen-presenting cells. Such mechanisms have been described for maltodextrin- and lipid-based nasal nanoparticles [24] and are consistent with the robust systemic IgY responses that were observed in this study. Avian colibacillosis typically progresses from respiratory or yolk sac infections to systemic septicemia (pericarditis, perihepatitis). A strong circulating IgY response is likely a key indicator of protective potential [11,39]. While VXN-EColi elicited robust systemic immunity and showed a favorable safety profile following in ovo administration, this study did not assess the protection against homologous or heterologous APEC challenge. The controlled challenge studies will be necessary to determine whether the observed immune responses translate into effective protection against clinical disease and bacterial colonization. While this study did not include direct quantitative comparisons with commercial colibacillosis vaccines, the robust systemic IgY responses that were observed suggest promising immunogenicity, and future head-to-head studies will be important to benchmark the nanovaccine against existing products.

To be viable for the poultry industry, a new vaccine must not interfere with mandatory immunization protocols. Our results prove that the co-administration of VXN-EColi with live-attenuated MDV, NDV, and IBDV vaccines did not diminish the antiviral antibody titers. This lack of interference is essential for the seamless integration of VXN-EColi into automated in ovo injection systems (such as INOVOJECT), which are currently used in over 90% of US hatcheries [40,41,42].

## 5. Conclusions

The increasing prevalence of multidrug-resistant APEC strains highlights the urgent need for alternative preventive strategies that can be applied early in life while reducing reliance on antibiotics. In this study, we demonstrate that NPL technology provides a flexible and safe platform for the development of a multivalent inactivated vaccine that is suitable for in ovo administration. The trivalent *E. coli* nanovaccine elicited strong systemic immune responses without compromising embryonic safety or post-hatch growth performance, addressing key constraints of early vaccination in poultry production. Unlike live-attenuated vaccines, this approach avoids the risk of reversion to virulence, reduces cold-chain dependency, and simplifies field handling. Importantly, in ovo administration of VXN-EColi did not interfere with concomitantly administered live-attenuated viral vaccines, supporting its compatibility with existing vaccination programs and co-administration strategies. Although protective efficacy remains to be confirmed in challenge models, future studies are warranted to evaluate VXN-EColi in controlled homologous and heterologous APEC challenges, to assess long-term immune memory, and to compare its performance directly with commercial colibacillosis vaccines. These results support NPL-based nanovaccines as a promising tool for early-life immunization and as a foundation for future applications of nanotechnology in poultry.

## Figures and Tables

**Table 1:** The description of the experimental groups.

Antiviral Vaccination	APEC Vaccine	*n*
G1 Newxxitek™ HVT + ND and BDA BLEN	no	350
G2 Newxxitek™ HVT + ND and BDA BLEN	VXN-EColi 1:0.3	350
G3 Newxxitek™ HVT + ND and BDA BLEN	VXN-EColi 1:0.5	350
G4 Newxxitek™ HVT + ND and BDA BLEN	VXN-EColi 1:1	350

**Table 2:** The egg parameters. The hatchability of fertile eggs (*n* = 1000) calculated as the ratio of all the chicks that hatched per the number of eggs that received in ovo vaccine administration and the average egg weight (*n* = 1000) and overall survival (*n* = 350) calculated as the difference between the initial number of birds in each group and the number of birds sacrificed at the end of the experiment (D42).

Vaccination	Number of Injected Fertile Eggs	Hatchability of Fertile Eggs (%)	Average Egg Weight (g)
G1 Newxxitek™ HVT + ND and BDA BLEN	1095	94.43	46.2
G2 Newxxitek™ HVT + ND and BDA BLEN + VXN-EColi 1:0.3	1045	95.82	47.5
G3 Newxxitek™ HVT + ND and BDA BLEN + VXN-EColi 1:0.5	1057	94.99	46.85
G4 Newxxitek™ HVT + ND and BDA BLEN + VXN-EColi 1:1	1071	94.13	48.5

**Table 3:** The overall survival (*n* = 350) of vaccinated chicks calculated as the difference between the initial number of birds in each group and the number of birds sacrificed at the end of the experiment (D42).

Vaccination	Initial Number of Chicks	Number of Chicks at D42	Survival at D42 (%)
G1 Newxxitek™ HVT + ND and BDA BLEN	350	330	94.29
G2 Newxxitek™ HVT + ND and BDA BLEN + VXN-EColi 1:0.3	350	330	94.29
G3 Newxxitek™ HVT + ND and BDA BLEN + VXN-EColi 1:0.5	350	335	95.71
G4 Newxxitek™ HVT + ND and BDA BLEN + VXN-EColi 1:1	350	339	96.96

## Data Availability

The raw data supporting the conclusions of this article will be made available by the corresponding author on request.
